# Left Supraclavicular Spindle Cell Lipoma

**DOI:** 10.1155/2010/942152

**Published:** 2010-05-25

**Authors:** Oladejo Olaleye, Bertram Fu, Ram Moorthy, Charles Lawson, Myles Black, David Mitchell

**Affiliations:** ^1^Department of ENT, William Harvey Hospital, Ashford, Kent TN24 0LZ, UK; ^2^William Harvey Hospital, Ashford, Kent TN24 0LZ, UK

## Abstract

*Background*. Spindle cell lipoma (SCL) is a benign lipomatous tumour, typically occurring in the posterior neck, shoulder or upper back of elderly males. They compose of fat, CD34 positive spindle cells, and ropey collagen on a myxoid matrix. This case highlights a rare presentation of SCL and the need for pre-operative diagnosis. *Case Report*. A 63-year-old gentleman presented with a pre-existing left supraclavicular mass that had recently increased in size. FNA and CT Scans were performed and results discussed in the mutidisciplinary team meeting. Excisional biopsy was recommended. *Radiology*. CT neck showed a left supraclavicular mass of fatty density with fine internal septations. A low-grade liposarcoma could not be excluded. *Histopathology*. FNA was indeterminate. Histology of specimen showed bland spindle cells with no evidence of malignancy. Immuno-histochemistry showed SCL with CD34 positivity and negative staining on CDK4 and p16. *Management*. Excision biopsy of the mass was performed which was technically difficult as the mass invaginated around the brachial plexus. The patient recovered well post-operatively with no neurological deficits. *Conclusion*. Spindle cell lipoma is a rare benign tumour and a pre-operative diagnosis based on the clinical context, imaging and immuno-histochemistry is crucial to management.

## 1. Introduction

Lipomasare slow growing fatty tumours of mesenchymal origin and can occur in any organ of the body. Spindlecell lipomas (SCLs) are lipoma variants which have the potential of neuromuscular invasion despite their benign histopathology. 

 This case report highlights a spindle cell lipoma variant in the supraclavicular area found intraoperatively to invade both the paravertebral muscles and the brachial plexus. Computerised tomography (CT) and Magnetic Resonance Imaging (MRI) findings were suggestive of possible malignancy but the biopsy and immune-histochemical stains confirmed the diagnosis as SCL. 

 This report highlights the need for making a preoperative diagnosis of spindle cell lipoma. This ensures accurate patient consent and appropriate preoperative surgical planning including surgical approach and extent of surgery.

## 2. Case Report

A-sixty-three-year-old gentleman presented with a two-year history of a left supraclavicular mass which had increased in size over the previous three months prior to presentation. The swelling caused discomfort with movement but there were no neurological deficits. He was a smoker with a past medical history of hypertension and type 2 diabetes mellitus. 

 On examination, there was a large tennis ball sized swelling in the left neck level V area with no palpable cervical lymphadenopathy. This swelling was smooth and nontender on palpation. ENT examination was normal. 

**Figure 1:**
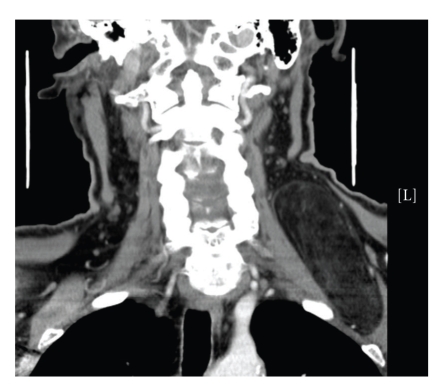
Coronal CT scan of the neck showing a left supraclavicular mass with internal septations.

**Figure 2:**
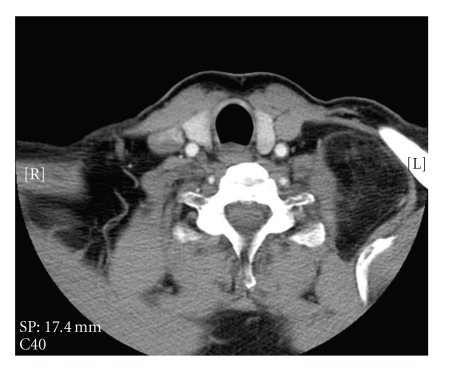
Axial CT scan of the neck showing extension of left supraclavicular mass posteriorly.

 Fine Needle Aspiration (FNA) of the mass was performed but it was nondiagnostic. CT neck showed a 46 × 69 × 91 mm left supraclavicular mass posterior to the sternocleidomastoid muscle and superficial to the paravertebral muscles, of fatty density with fine internal septations (Figures 1 and 2). The radiological appearance was suggestive of a lipoma but a low-grade liposarcoma could not be excluded due to the internal complexity confirmed on MRI. A CT of the chest was normal. 

 The case was discussed at the multidisciplinary meeting and due to the suspicion of malignancy, and a nondiagnostic FNA, excision biopsy was recommended. The operation was technically difficult because the mass invaginated around the brachial plexus and extended posteriorly through the prevertebral fascia. Complete excision of the mass was undertaken with mobilisation and preservation of the brachial plexus. The patient made a full recovery with no neurological deficits.

## 3. Pathology

The specimen weighed 132  grams and consisted of well-circumscribed fatty tissue. Histological examination showed mature adipocytes interspersed with fibrous septa containing bland spindle cells with focal pseudo-angiomatous arrangement. There was the presence of ropey collagen, a very characteristic finding in spindle cell lipoma. No lipoblasts were seen and the spindle cells showed strong uniform immuno-histochemical positivity for CD34. 

 Subsequent review also showed negative staining for CDK4 and p16, supporting the diagnosis of spindle cell lipoma, and excluding atypical lipomatous neoplasm.

## 4. Discussion

Spindle cell lipoma (SCL) is a benign tumour which typically occurs on the posterior neck, shoulder, or upper back in predominantly elderly males. There is a marked male predilection especially in the sixth decade which may be partly explained by androgen receptors reactivity [1]. Other rare reported sites of occurrence in the head and neck region include the face [2], suprasellar region [3], buccal fat pad [4], oesophagus [5], nasal vestibule [6], the tongue [7, 8], floor of mouth [9], vallecula [10] and parotid gland [11]. 

**Figure 3:**
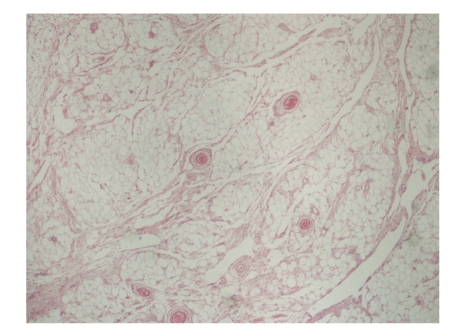
Low-power view of the tumour. Islands of adipocytes are surrounded by loose fibrous septa with artefactual clefts giving a pseudo-angiomatous appearance (H&E x20).

**Figure 4:**
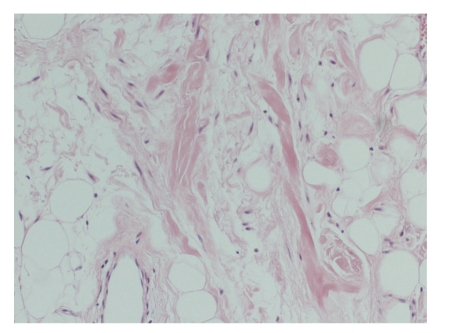
High-power view of fibrous area with ropey collagen bundles and scattered spindle cells (H&E x100).

**Figure 5:**
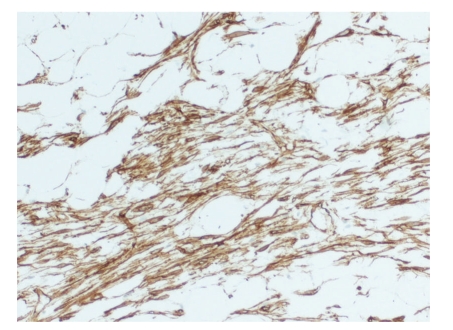
High-power view of strong uniform staining of spindle cells with antibody to CD34 (QBEND 10) (x100).

**Figure 6:**
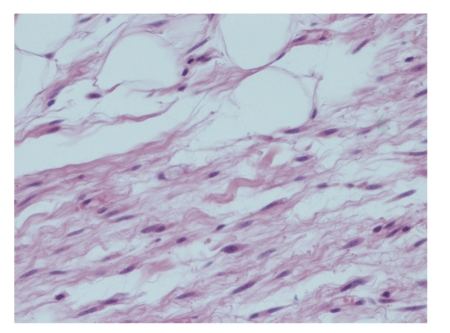
High-power showing spindle cells arranged in parallel fascicles within collagenous stroma (H&E x100).

**Figure 7:**
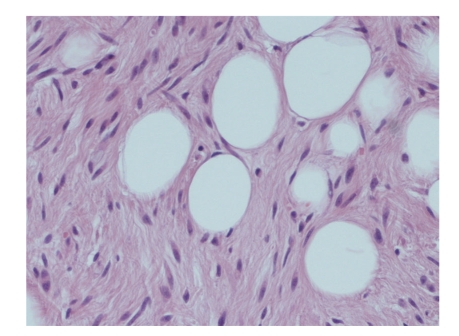
High-power view of more haphazard arrangement of spindle cells within fibrous stroma and mixed with adipocytes (H&E x100).

 Histologically it consists of a proliferation of mature adipocytes, collagen fibres, and spindle cells within a myxoid stroma (Figure 3, 4, 5, 6, and 7) and can infiltrate between skeletal muscle fibres [2]. On immuno-histochemistry, the spindle cells express CD34 and they are negative for S-100 protein. They also characteristically express losses of chromosomes 13q and/or 16q [4]. They are however desmin negative tumours and this may help differentiate them from other CD34 positive tumours [12].

## 5. Differential Diagnoses

Differential diagnoses include atypical lipomatous tumour/well-differentiated liposarcoma, pleomorphic lipoma, neurofibroma, nuchal fibroma, lipoblastoma, hibernoma, cellular angiofibroma [13], extramammary myofibroblastoma, and solitary fibrous tumour. 

 It is important to note that reactivity to CD34 immuno-histochemical stain is not specific to only spindle cell lipoma as other entities can demonstrate immunoreactivity to CD34 such as atypical lipomatous neoplasm (ALN)/well-differentiated liposarcoma (WDL). 

 ALN/WDL is the most common form of liposarcoma seen in late adult life with equal gender affectation generally. They contain a significant component of mature fat and tend to have less well-defined borders than lipomas [14]. The favoured sites for ALN/WDL are deep soft tissues of extremities and retroperitoneum compared to SCL that favour the subcutis of the posterior neck, back and shoulders. 

 SCL shows cytogenetic aberrations—mostly loss of 16q material and less frequently material from 13q. This coupled with the usual absence of giant marker and ring chromosomes that are typically seen in atypical lipomatous tumour/well-differentiated liposarcoma supports their distinction. 

 Immunostaining for MDM2 and CDK4 has been shown to be a relatively sensitive and specific means of identifying and separating atypical lipomatous neoplasm/well-differentiated liposarcoma from various benign lipomatous lesions [15, 16]. 

 In this case report, histologic findings did not demonstrate sufficient nuclear atypia to suggest a diagnosis of atypical lipomatous tumour and further immuno-histochemical markers that would make such a diagnosis possible (CDK4, p16) were found to be negative. 

 SCL shares overlapping clinical, morphologic, and immuno-histochemical findings with pleomorphic lipoma as well as the same cytogenetic aberrations. The differential diagnosis of SCL or pleomorphic lipoma depends on which elements predominate [14]. 

 Dermatofibrosarcoma protuberans (DFSP) is also a differential of SCL. It however typically arises in the dermis, often in younger patients and lacks the characteristic ropey collagen of SCL [14]. 

 Schwannoma and neurofibroma have a similar striking nuclear palisading and conspicuous mast cell infiltrate present in some SCL. These however invariably express S-100 protein which is negative in SCL [14]. 

 SCL may also be mistaken for several sarcomas, including myxoid liposarcoma or spindle cell liposarcoma. SCL is however more circumscribed, lacks lipoblasts, with characteristic ropey collagen bundles. The presence of multinucleated floret-like giant cells is characteristic of pleomorphic lipoma and is occasionally seen in atypical lipomatous tumour/well-differentiated liposarcoma. 

 In this case report, the spindle cell lipoma presented a diagnostic and management dilemma as it could not be clinically differentiated from a liposarcoma which would require a more radical approach to treatment. The radiologic findings of a complex internal architecture with fine septations allowed differentiation of the preoperative differential diagnosis from a benign lipoma or possible malignancy but could not differentiate between a liposarcoma or a spindle cell lipoma. 

 The authors suggest that when radiologic findings suggest a complex internal architecture suspicious for a diagnosis of a possible lipomatous tumour, it is imperative to obtain a biopsy prior to definitive surgery. Histologic interpretation is crucial and this can be supported by the use of immune-histochemical stains. Immuno-histochemical stains demonstrating positivity for CD34 can be helpful in diagnosis of CD34 positive tumours such as spindle cell lipoma, and the differential diagnoses discussed such as atypical lipomatous tumour and dermatofibrosarcoma protuberans (DFSP). As discussed, further differentiation can be made based on clinical presentation, immunoreactivity for actin or desmin, S-100 protein staining of mature lipocytes, the presence/absence of ropey collagen bundles, multinucleated floret-like giant cells and cytogenetic aberrations. In difficult cases, fluorescence in situ hybridization (FISH) analysis can be helpful. 

A preoperative diagnosis of spindle cell lipoma is crucial to surgical management as it impacts on the consent process, surgical approach, extent of surgery, and management. 

 In making a preoperative diagnosis, tissue sampling is required with either FNA or core biopsy. However, it is important to note that FNA may be nondiagnostic as it was in this case, because the specimen consisted largely of fatty cells. Core biopsy is preferable and can be obtained by an initial procedure under anaesthesia prior to definitive surgery based on a histological diagnosis. A preoperative diagnosis will guide the consent process as such patients can be warned of the risks of neurovascular injury as well as determining the extent of surgery.
